# Mitochondrial Mass Assessment in a Selected Cell Line under Different Metabolic Conditions

**DOI:** 10.3390/cells8111454

**Published:** 2019-11-18

**Authors:** Anna Costanzini, Gianluca Sgarbi, Alessandra Maresca, Valentina Del Dotto, Giancarlo Solaini, Alessandra Baracca

**Affiliations:** 1 Laboratory of Biochemistry and Mitochondrial Pathophysiology, Department of Biomedical and Neuromotor Sciences, University of Bologna, 40126 Bologna, Italy; anna.costanzini2@unibo.it (A.C.); gianluca.sgarbi@unibo.it (G.S.); 2 Department of Medical Sciences, University of Ferrara, 44121 Ferrara, Italy; 3 UOC Clinica Neurologica, IRCCS Istituto delle Scienze Neurologiche di Bologna, 40126 Bologna, Italy; alessandramaresca@gmail.com; 4 Unit of Neurology, Department of Biomedical and Neuromotor Sciences, University of Bologna, 40139 Bologna, Italy; vale.deldotto@gmail.com

**Keywords:** mtRFP, mitochondrial mass, hypoxia, resveratrol, flow cytometry, cancer cells

## Abstract

Changes of quantity and/or morphology of cell mitochondria are often associated with metabolic modulation, pathology, and apoptosis. Exogenous fluorescent probes used to investigate changes in mitochondrial content and dynamics are strongly dependent, for their internalization, on the mitochondrial membrane potential and composition, thus limiting the reliability of measurements. To overcome this limitation, genetically encoded recombinant fluorescent proteins, targeted to different cellular districts, were used as reporters. Here, we explored the potential use of mitochondrially targeted red fluorescent probe (mtRFP) to quantify, by flow cytometry, mitochondrial mass changes in cells exposed to different experimental conditions. We first demonstrated that the mtRFP fluorescence intensity is stable during cell culture and it is related with the citrate synthase activity, an established marker of the mitochondrial mass. Incidentally, the expression of mtRFP inside mitochondria did not alter the oxygen consumption rate under both state 3 and 4 respiration conditions. In addition, using this method, we showed for the first time that different inducers of mitochondrial mass change, such as hypoxia exposure or resveratrol treatment of cells, could be consistently detected. We suggest that transfection and selection of stable clones expressing mtRFP is a reliable method to monitor mitochondrial mass changes, particularly when pathophysiological or experimental conditions change ΔΨ_m_, as it occurs during mitochondrial uncoupling or hypoxia/anoxia conditions.

## 1. Introduction

Mitochondria play a pivotal role in cellular homeostasis mainly by providing energy, regulating the redox state of the cell, the calcium homeostasis, releasing signaling messengers, and in many circumstances inducing cell death. To ensure healthy and functional mitochondria mass, cells continuously adjust the number, size, and shape of mitochondria in response to stress conditions and metabolic needs [1,2]. The architecture and interconnection of the mitochondrial network are constantly modified by fusion and fission events, and cooperating with the mitochondrial quality control systems to implement the turnover of the organelles, removing superfluous or damaged mitochondria and stimulating the biogenesis of the new ones [1,3]. Increased interconnection of the mitochondrial network by enhanced fusion has been related to increased oxidative phosphorylation (OXPHOS) efficiency and energy supply by determining the functional cooperation among mitochondria [4]. Fusion of organelles within the same cell also compensates for the effect of detrimental mutation of the mitochondrial genome by mixing mtDNA and maintaining the degree of heteroplasmy below the pathogenic threshold. Together with fission, fusion regulates the exchange and mixing of proteins and lipids among mitochondria, a mechanism that mitigates the structural and functional damage of organelles determined by pathological conditions, ageing, and environmental insults, thus limiting the cellular redox stress and apoptosis activation [5,6]. When the efforts of the cells to repair and to rescue injured mitochondrial component are insufficient to recover organelles homeostasis, the activation of stress-response pathways can lead to the exposure of “eat-me” signals on the surface of the organelle, which are able to trigger their specific degradation via mitophagy [7]. Among the mitophagic pathways, PINK1/Parkin activation was described in pathologic conditions characterized by the collapse of the mitochondrial membrane potential (ΔΨ_m_), as in ischemia [8,9]. Moreover, some constitutive proteins of the outer mitochondrial membrane (OMM), such as BNIP3, BNIP3L, and FUNDC1, can activate mitophagy and their expression and activation have been reported to be reliant on chronic hypoxia and HIF-1α stabilization, therefore mediating the mitochondria clearance in cells with limited oxygen availability [10,11,12]. Alterations in nutrient availability, temperature, and molecular stimuli that determine the activation of hormones and growth factor-initiated intracellular signaling pathways enhance the expression of the master regulators of the mitochondrial biogenesis PGC-1α and PGC-1β. Increased levels of those transcriptional coactivators were related to the increase of both mitochondrial mass and mitochondrial activities, including ROS-scavenging enzymes, OXPHOS components, metabolic pathways, import complexes, fission and fusion proteins, and mitochondrial sirtuins [13,14,15]. Dysregulation of mitophagy and mitochondrial biogenesis and in turn mitochondria mass have been related to many pathological conditions, therefore, the evaluation of both mitochondrial mass and network interconnection have become crucial to understanding the mechanisms of cell adaptation to pathological conditions and to specific therapies.

Fluorescent probes such as tetramethylrhodamine methyl ester (TMRM), nonylacridine orange (NAO), and MitoTrackers are commonly employed to investigate mitochondria mass and dynamics [16]. Unfortunately, the reliability of measurements with exogenous probes is hampered by the efficiency of dye internalization in cells and mitochondria. Depending on their chemical peculiarity, the accumulation of probes in mitochondria is affected by a number of parameters, including ΔΨ_m_, membrane composition, and cell redox state. These parameters are commonly altered by drug treatments and stress conditions reproduced in experimental investigations [17,18,19]. Over recent years, recombinant fluorescent proteins genetically encoded by cells and selectively addressed to the cellular district of interest have been used as reporters for a plethora of structural and functional analysis in cells and animal models [20]. The constitutive expression of the fluorescent protein reporter and its specific localization within the cells by inserting a targeting signal to the protein sequence, could overcome the limitations of the internalization efficiency, typical of exogenous fluorescent probes. Fluorescent proteins specifically targeted to mitochondria (mtFPs) are currently employed to follow mitochondrial dynamics and network organization in living cells (see reference [21] for a basic review). Among others, the mtFP approach to monitoring quantitative changes of mitochondria content in living cell by microscopy and flow cytometry were recently reported [22]. However, to our knowledge, a clear-cut demonstration of the correlation existing between the fluorescence intensity of the mtFP reporters and the mitochondrial mass of cells has not been described. The aim of the present work is to both contribute a guideline to produce trustable cellular models stably expressing a fluorescent protein targeted to mitochondria that save the main structural and functional characteristics of the parental cell line, and prove that the mtFPs approach is a reliable method for quantifying mitochondrial mass changes in cells.

## 2. Materials and Methods

### 2.1. Cell Cultures

Human osteosarcoma 143B cell lines and all the clones were cultured in Dulbecco’s Modified Eagle Medium (DMEM) supplemented with 10% fetal bovine serum, 25 mM glucose, 4 mM glutamine, and 1 mM pyruvate, in the presence of 100 U/mL penicillin, 100 µg/mL streptomycin, and 0.25 µg/mL amphotericin B (complete medium). Cells were maintained at 37 °C in a humidified atmosphere with 5% CO_2_. Cell culture reagents were purchased from Gibco (Thermo Fisher Scientific; Walthman, MA, USA), with the exception of glucose, glutamine and pyruvate, which were provided by Sigma-Aldrich (Saint Louis, MO, USA).

### 2.2. Generation of mtRFP-Positive Osteosarcoma Clones

Stable clones were obtained from 143B parental cells by transfection with the pcDNA3.1 plasmid expressing a mitochondrially targeted dsRED fluorescent protein (mtRFP). pcDNA3.1 plasmid expressing a dsRED fluorescent protein fused to the cleavable targeting sequence of subunit VIII of cytochrome c oxidase (COXIII) was a kind gift from Dr M. Zaccolo (Venetian Institute of Molecular Medicine, Padova, Italy). Transfections were performed according to Barbato et al. [23] using a 2 µg plasmid DNA and DNA:PEI ratio of 1:8 (w/w). After 48 h, cells were detached with trypsin and cloned by limiting dilution method in complete medium supplemented with 500 µg/mL Geneticin (G418). Clones with similar mtRFP fluorescence intensity were selected and maintained in 100 µg/mL G418 as continuous cell lines. Cells were seeded at the optimal density and maintained in complete medium without G418 for 24 h before starting the experiments.

### 2.3. mtRFP-Fluorescence Intensity Assay

In order to evaluate whether intracellular fluorescence quenching phenomena occurred, we compared the mtRFP fluorescence intensity measured in intact cells with the fluorescence measured upon cell lysis using a Jasco FP-550 spectrofluorometer. Briefly, cells expressing mtRFP were resuspended in Hank’s Balanced Salt Solution (HBSS) at final concentration of either 1 or 2 × 10^6^ cells/mL, and the fluorescence intensity was detected by fluorometry at λ_ex_ 556 and λ_em_ 575 nm. The same samples were then incubated for 3 min with 0.2% Triton X-100 (Sigma-Aldrich, St. Louis, MO, USA) to obtain a complete lysis of mitochondria, allowing mtRFP to dilute within the whole solution, and the fluorescence intensity was recorded again. The mtRFP fluorescence detected at 575 nm was similar under the two conditions tested.

### 2.4. Assessment of mtRFP Fluorescence Changes Following Mitochondrial Mass Modulation

The experiments were performed seeding the cells at a density of 30,000 cells/cm^2^ in complete medium, which was replaced at the beginning of the experiments (t = 0). To evaluate whether the mtRFP signal is a useful parameter to measure mitochondrial mass changes, we exposed cells to either hypoxia or resveratrol (RSV). Our previous data showed that cells exposed to hypoxia reduced significantly the mitochondrial mass [24], whereas it was demonstrated that culturing cells in the presence of micromolar concentrations of RSV induces an increase of the mitochondrial mass [25,26]. Culture hypoxia conditions were achieved using the INVIVO_2_ 200 hypoxic workstation (Ruskin Technology Ltd., Bridgend, South Wales, UK), whilst RSV treatment was performed by culturing cells in the presence of an increasing concentration of the polyphenol (1, 5, 10, and 15 µM). At the end of the experiments, cells were either imaged by fluorescence microscopy or detached and processed for both the citrate synthase (CS) activity assay and the cytofluorometric assessment of the mtRFP fluorescence intensity.

### 2.5. Flow Cytometry Measurements of Fluorescence Intensity within mtRFP-Positive Cells

The fluorescence intensity of clones stably expressing mtRFP was measured by cytometry (Muse cell analyzer, Millipore, Burlinghton, MA, USA), using an excitation wavelength at 532 nm and detection at 576/28 nm, as previously reported with more details [27]. Data were analyzed using the Flowing software 3.1 (Cell Imaging Core, Turku center for Biotechnology, University of Turku, Turku, Finland).

### 2.6. Fluorescence Microscopy

Fluorescence images of mtRFP-positive osteosarcoma cells were acquired using a fluorescence inverted microscope (Olympus IX50 equipped with a CCD camera) according to Sgarbi et al. [28]. Multiple high-power images were acquired (40×) with IAS2000 software (Delta Sistemi, Rome, Italy). Fluorescence micrographs of mtRFP-expressing cells were acquired using a specific set of filters: excitation 540/20 and emission 610/40. At least 10 different optic fields were acquired for each experimental condition tested.

### 2.7. Citrate Synthase Activity

Citrate synthase activity was evaluated as an index of mitochondria content in cells and it was assayed according to the spectrophotometric method described by Trounce et al. [29]. Cells were detached, washed in HBSS, and the assay was performed using the method that we previously described [30]. Protein concentrations of the samples were determined by a modified protocol of the Lowry procedure as reported [31].

### 2.8. Mitochondrial DNA Quantification

Total DNA was isolated from cells using the NucleoSpin tissue kit (Macherey & Nagel, Duren, Germany), following manufacturer’ instructions. Mitochondrial DNA (mtDNA) content was assessed by real time-PCR through absolute quantification, as previously described [32].

### 2.9. Respiration Measurements

The oxygen consumption rate was measured in permeabilized cells (60 μg/mL digitonin) at 30 °C with 10 mM glutamate/10 mM malate (plus 1.8 mM malonate) as substrate, using an oxygen Clark-type electrode as previously reported by Bosetti et al. [33]. State 4 and State 3 respiration rates were measured in the absence and in the presence of 0.5 mM ADP, respectively. The respiratory control ratio (RCR) is defined as the State 3/State 4 respiratory rate ratio.

### 2.10. Statistical Analysis

Statistical analyses were performed using OriginPro 8.5 software, choosing the most appropriate test. Statistical significance was declared at *p* < 0.05. Comparisons among multiple groups were made by a One-Way repeated measures analysis of variance (ANOVA) followed by Dunnett’s post hoc test. Data are presented as means ± SD.

## 3. Results

### 3.1. The mtRFP Fluorescence Is Stable in Osteosarcoma Transfected Cells

**Figure 1:**
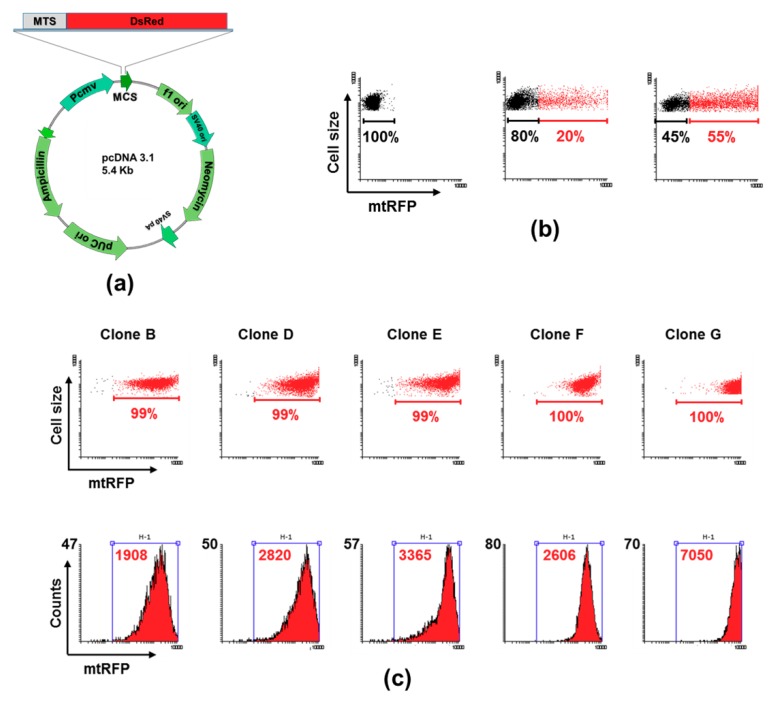
Preparation and isolation of mtRFP clones from 143B cells. (**a**) Scheme of pcDNA3.1 plasmid used to transfect cells, showing the mitochondrial targeting sequence (MTS) of COX VIII attached to a dsRED (RFP) sequence. (**b**) Representative dot plot graphs, obtained by flow cytometry, displaying the mtRFP fluorescence intensity of untreated (left panel), 24 h (middle panel) and 48 h (right panel) transfected cells. The percentage of mtRFP-positive cells is indicated in red. (**c**) Analysis of single clones prepared by limiting dilution. Top panel: dot plot analysis showing percent of mtRFP-positive cells (red). Bottom panel: histogram representation of the dot plots analysis showing the cell fluorescence distribution; the mean fluorescence intensity of H1-gated population is indicated in red.

To prepare stably-expressing mitochondrially targeted RFP clones, 143B osteosarcoma cells were transfected with the pcDNA3.1-mtRFP plasmid (Figure 1a). The transfection efficiency was evaluated by flow cytometry and some 55% of 143B cells had positive results in response to mtRFP expression after 48 h transfection (Figure 1b). Notably, the COX VIII subunit targeting sequence leads the red fluorescent protein into the mitochondrial matrix [34]. Cells were then cloned in the presence of G418 to obtain stable clones expressing mtRFP; different clones were selected and screened to assay the mean fluorescence intensity of the cell populations. Among several clones showing different mean fluorescence intensities (Figure 1c), clones D and E displaying similar growth rates and mean fluorescence intensities were chosen. In addition, clone G characterized by the fluorescence intensity nearly double that of clones D and E, was also considered in the following experiments.

**Figure 2:**
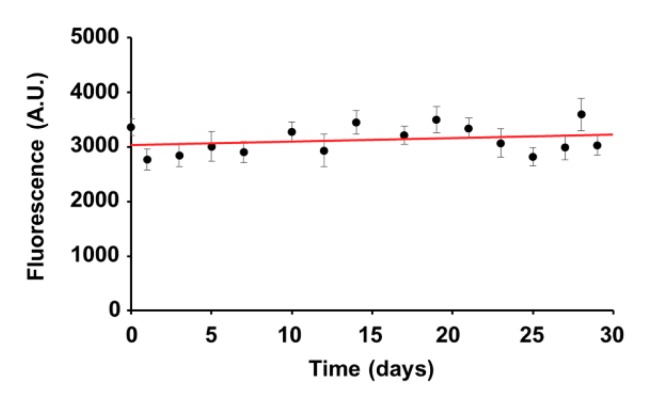
Stability of mtRFP fluorescence intensity in osteosarcoma derived clones. Representative time dependence of the mean fluorescence intensity assayed in mtRFP-positive cells (143B-Clone E) over a month. Linear regression (red line) of the data show that the mean florescence intensity of mtRFP-positive cells was stable.

First, the fluorescence stability of the selected mtRFP-expressing clones over a month was examined. The expression of the mtRFP was checked by assessing the mean fluorescence intensity of each clone every other day when cells were split. In this time frame, all the clones maintained similar mean fluorescence intensity, showing moderate and not significant oscillations (generally not exceeding 10%). As an example, the fluorescence intensity trend of the 143B-Clone E is shown in Figure 2.

### 3.2. The mtRFP Fluorescence Intensity Is Linked to the Cell Mitochondria Mass and It Is not Affected by Quenching Phenomena

**Figure 3:**
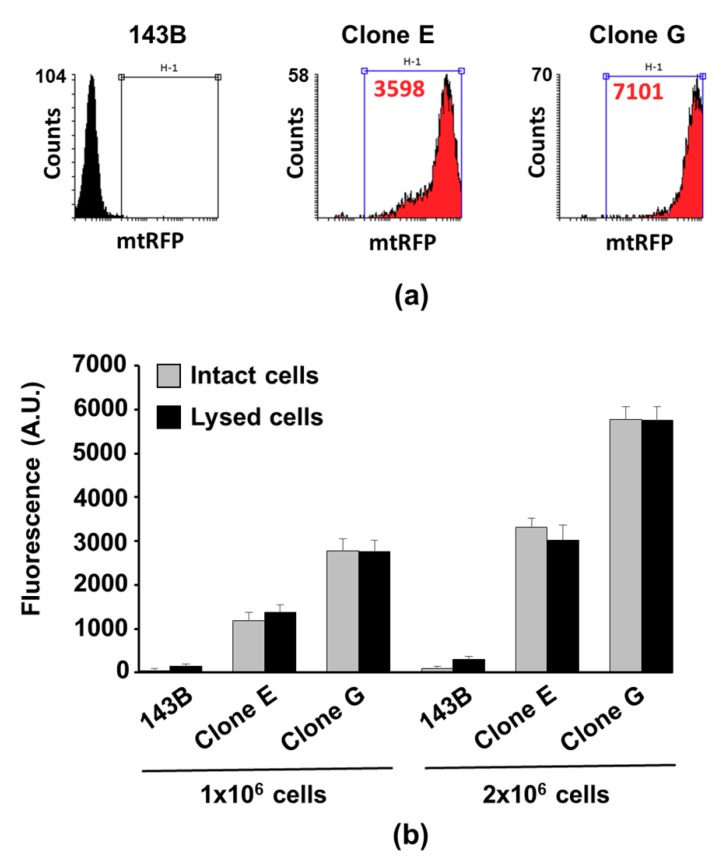
mtRFP fluorescence intensity and mitochondria mass are directly related. (**a**) Cell fluorescence distribution of both 143B parental cells and derived mtRFP clones (143B-Clone E and the 143B-Clone G); the mean fluorescence intensity of H1-gated population is indicated in red. (**b**) Fluorometric measurements of two different amounts of intact and lysed mtRFP-cells.

Fluorescence quenching phenomena are frequently detected in assays of probes used in intact cells; quenching mainly occurs by energy transfer from the excited fluorophore to other fluorophores or by interaction with quenching molecules in the proximity. Therefore, the fluorescence dissipation may be particularly significant in samples where the fluorophore is present at high concentration or where the fluorophore is limited within a small cellular compartment, as mitochondria are [35,36]. To avoid the underestimation of the fluorescence intensity and, in turn, the incorrect quantification of the mitochondrial mass, possible mtRFP fluorescence quenching was examined under different experimental conditions. Thus, fluorometric assessments were performed by using two mtRFP-positive clones (143B-Clone E and 143B-Clone G), showing mean fluorescence intensities that were significantly different: 3598 A.U. and 7050 A.U., respectively (Figure 3a). The osteosarcoma 143B parental cell line was used as a negative control. The cell fluorescence distribution of samples containing either 1 × 10^6^ or 2 × 10^6^ cells was analyzed by fluorometry and then immediately treated with Triton X-100 in order to allow the release of the red fluorescence proteins from permeabilized mitochondria. Notably, the mean fluorescence intensity of the lysed samples was comparable to that of the intact cells (Figure 3b), allowing us to exclude the occurring of quenching phenomena caused by high probe concentration inside mitochondria. In addition, the quantitative relationship between the average fluorescence intensities of 143B-Clone E and 143B-Clone G determined by flow-cytometry was maintained independently of the number of cells in the samples (1 × 10^6^ or 2 × 10^6^ cells). Furthermore, the “linearity” of the mtRFP method found support by the doubling of the mean fluorescence intensity of samples containing 2 × 10^6^ cells compared to the intensity detected in samples containing a half number of cells of the same clone (Figure 3b). Altogether, these data proved that the cell mtRFP signal was linear within the conditions chosen, being dependent on the mtRFP protein content, and excluded the occurrence of quenching phenomena.

### 3.3. Expression of mtRFP Did not Alter Mitochondrial Functions

**Figure 4:**
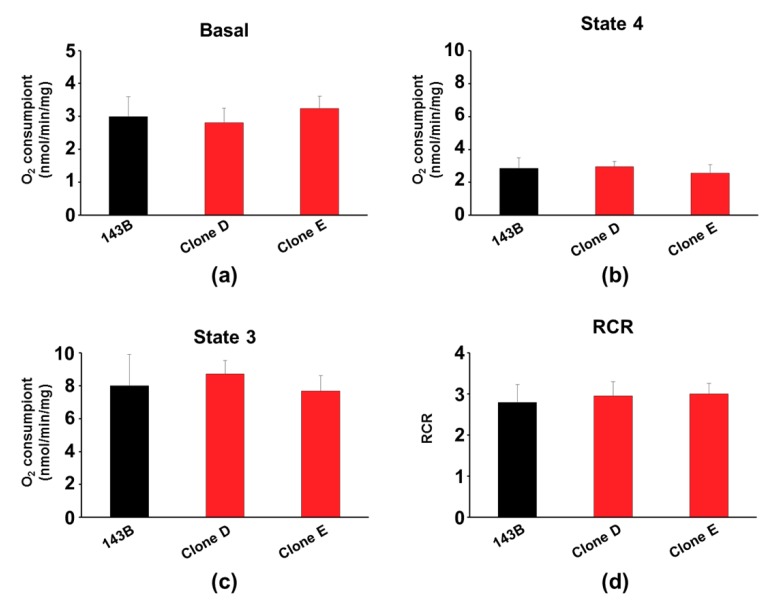
Oxygen consumption rate of 143B and mtRFP-expressing cells. Cell respiration of the parental 143B (black bar) and mtRFP-expressing cells (red bars) was assayed in intact cells (**a**) or in digitonin-permeabilized cells under both Complex I-driven state 4 (**b**) and state 3 (**c**) respiration rates, expressed as nmol O_2_/min/mg protein. From b and c, the respiratory control ratio (RCR) was calculated (**d**). Histograms show the mean ± SD of three different experiments.

To verify whether the transgenic expression of mtRFP could interfere with mitochondrial functions and, in particular, with the OXPHOS activity, we measured the oxygen consumption rate in both intact and digitonin-permeabilized cells. No significant changes were detected in the basal (intact cells), state 4 and state 3 respiration of both 143-Clone D and 143B-Clone E compared to parental 143B cells (Figure 4a–c). Therefore, the RCR, an index of the intactness of the OXPHOS machinery, was also unaffected (Figure 4d), Overall, the presence of an exogenous fluorescent protein in mitochondria did not alter the mitochondrial respiratory chain or the OXPHOS system.

### 3.4. The mtRFP Fluorescence Intensity Correlates with the Citrate Synthase Activity

**Figure 5:**
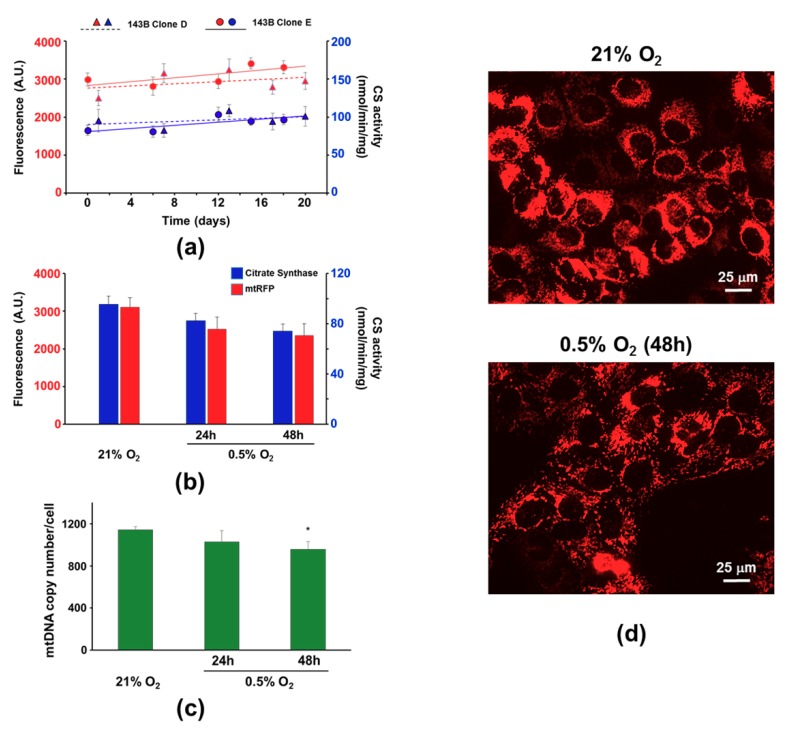
Mitochondria mass evaluation of 143B-Clones D and E. (**a**) mtRFP fluorescence intensity (red lines) and CS activity (blue lines) assayed during 18 days in Clone D (triangles) and Clone E (circles). Points are the mean ± SD of four assays. (**b**) Mean fluorescence intensity (red bars) and CS activity (blue bars) assayed in Clones D and E cultured in both normoxic (21% O_2_) and hypoxic conditions (0.5% O_2_). (**c**) mtDNA content evaluated in Clone E exposed to both normoxia and hypoxia. Histograms show the mean ± SD of three different experiments. * *p* < 0.05 and ** *p* < 0.01 indicate the statistical significance of data. (**d**) Representative fluorescence microscopy images (magnification 40×) of Clone E cultured 48 h either in hypoxia (bottom panel) or control conditions (top panel).

To evaluate whether the signal of the mtRFP could be considered an index of the cell mitochondria content, the fluorescence intensity trend of the 143B-Clones D and E was compared with their own CS activity over a period of 18 days. At each established time, the cells at 90% confluence were detached and assayed for both cytofluorometric analysis of mtRFP fluorescence intensity and CS activity, whereas a fraction was re-seeded for the culturing. The fluorescence and the citrate activity changed in parallel. For an easy comparison, both the mean fluorescence intensity and the CS activity were plotted in a double y-axis graph (Figure 5a).

A second experiment was carried out culturing 48 h the 143B-Clones D and E either in normoxia (21% O_2_) or hypoxia (0.5% O_2_): the latter condition could decrease the cell mitochondrial mass through the activation of autophagy, as we recently showed [24]. Upon 24 and 48 h hypoxia exposure, the cells were detached and samples were collected to assay both the mtRFP fluorescence intensity by flow cytometry and the CS activity. For an easy comparison, the mean fluorescence intensity and the CS activities were plotted in a double y-axis graph (Figure 5b). Both parameters decreased by about 15%–20% at 24 h and 25% at 48 h hypoxia exposure compared to controls. To further validate the use of mtRFP fluorescence as an index of the mitochondrial mass, we evaluated the mtDNA copy number in cells exposed to hypoxia (Figure 5c). Increasing time of cell hypoxia exposure led to a progressive mtDNA content loss in 143B-Clone E: the nearly 20% decrease of mtDNA was statistically significant compared to control at 48 h. A decrease of mtRFP fluorescence could also be observed at a glance by fluorescence microscopy of adherent cells (Figure 5d).

**Figure 6:**
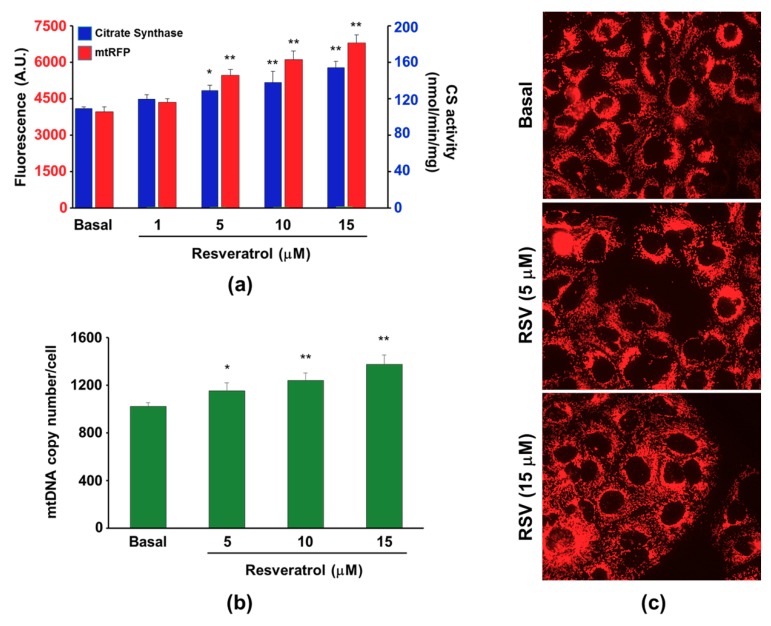
Mitochondrial mass evaluation in 143B-Clone E cells exposed for 48 h to increasing concentration of RSV. (**a**) Relationship between the mean fluorescence intensity values (red bars) and the CS activity (blue bars) measured in cells cultured in the absence or in the presence of increasing RSV concentration. (**b**) Cellular mtDNA content evaluated in the presence of different RSV concentrations. Histograms show the mean ± SD of three different experiments. * *p* < 0.05 and ** *p* < 0.01 indicate the statistical significance of data. (**c**) Representative fluorescence microscopy images (magnification 40×) of cells cultured in either the absence or presence of RSV.

Finally, stimulation of mitochondria biogenesis with increasing concentration of RSV (5–15 µM), a natural polyphenol capable to enhance both biogenesis and function of mitochondria [37], determined the enhancement of both CS activity and mtRFP fluorescence intensity (Figure 6a). Significantly, the change of the mtRFP fluorescence intensity was higher than that of the CS activity at any RSV concentration tested, indicating a higher sensitivity of the fluorescence method to monitor mitochondrial mass changes. Notably, at 1 to 5 µM RSV, the two parameters did not change significantly, as was shown recently in our laboratory [14]. Moreover, the assay of mDNA content confirmed the increase of mitochondrial mass observed in cells treated with RSV. Indeed, 48 h treatment of the 143B-Clone E with increasing RSV concentrations led to a significant progressive rise (from 10% to nearly 35%) of the mtDNA copy number. These mtDNA content changes quite closely matched the mtRFP fluorescence intensity increase when assayed under the same conditions (Figure 6b). Incidentally, the progressive increase of mtDNA content as the RSV concentration increased was similar to that measured by the CS assay. The mitochondria content enrichment induced by an increasing concentration of RSV was also appreciable by a simple fluorescence microscopy observation of the cells (Figure 6c). Incidentally, we ran the fluorescence emission spectra of mtRFP in the presence and in the absence of 15 µM RSV and did not observe significant differences. In addition, we tested whether RSV could affect the mtRFP in the presence of the cells and the assays did not show any significant interference (data not shown).

## 4. Discussion

The mitochondrial mass of cells is currently evaluated by means of one of the following methods: the biochemical method based on the assay of the matrix specific enzyme CS, the genetic method based on the measurement of the mtDNA copy number, or by evaluating the mass of typical mitochondrial proteins as translocase outer membrane (TOM) subunits through immunoblotting. Generally, biochemical methods are the most highly quantitative methods, but they require many µg of sample, are rather time-consuming, and it is inherently difficult to avoid interference in the assay when they are used. For instance the measurement of the CS activity, based on the use of the reagent 5,5’-dithio-bis (2-nitrobenzoic acid), may be affected by SH-containing molecules other than Coenzyme A, including those from cysteines and reduced glutathione [38]. The assay of the mtDNA is very expensive and possibly a quantitative real-time PCR must be available, therefore the use of fluorometric methods may represent a valid choice. Accordingly, we evaluated the potential use of mitochondrially targeted RFP to measure by flow cytometry changes of mitochondrial mass occurring in mtRFP+ cells exposed to both different environmental conditions and chemicals. We first demonstrated that the osteosarcoma clones stably expressing the red fluorescent protein within mitochondria retain structure and metabolic features of the cell line of origin.

To validate the method, mitochondrial mass changes induced in RFP+ cells by hypoxia exposure or RSV treatment were detected by mtRFP flow cytometry and compared to both CS activity and mtDNA content analysis. Incidentally, evaluation of mtDNA content increases have been recently correlated with variations in specific proteins as PGC-1a, NRF1, and TFAM that were directly associated with mitochondrial biogenesis in LHON patients [32]. Since similar results were obtained comparing different methods of analysis, we propose that mtRFP+ clones are reliable models to study processes associated with mitochondrial mass changes as they occur in mitophagy/autophagy or upon exposure of cells to starvation, hypoxia, or drugs.

We wish to highlight that the mtRFP-based method allows us to evaluate the mitochondrial mass changes in intact cells, providing the advantage of avoiding the uncertainties inherent to CS activity and mtDNA content analysis. Indeed, putative functional CS variations associated with metabolic changes caused by different cell culture conditions might lead to biased results [39]. In addition, the method here proposed might circumvent the potential under/over-estimation of the mtDNA content, due to the high cancer cell genomic instability [40,41] that could vary the copy number of the reference nuclear gene. Moreover, the mtDNA copy number might be affected by the redox state of the cells. Suliman et al. [42] reported that mtDNA quantity is affected by the mitochondrial GSH/GSSG indicating that the levels of ROS can modulate the mtDNA/nDNA. Notably, mitochondrial mass changes and/or imbalance of the fusion/fission processes occurring in mtRFP+ cells may be readily and easily captured by fluorescence microscopy. This qualitative information can be finished by a quantitative assay of the mitochondrial mass change by cytometry. Of course, to obtain correct data, it is important to verify the absence of quenching phenomena in the particular kind of cells examined and to verify the concentration of the mtRFP in order to avoid self-quenching [35,36]. To our knowledge, this is the first study demonstrating that different inducers of mitochondrial mass modulation such as hypoxia exposure and RSV treatment of cells can be used to evaluate the extent of the phenomenon. Differences may reflect the different steps of the autophagic signal transduction pathway induced by hypoxia or the stimulating capacity of RSV to promote mitochondrial biogenesis [43]. Therefore, mtRFP serves as a quantitative marker to analyze mitochondrial mass changes. However, a possible limitation of this method concerns the presence of mitochondrial import machinery defects in the cells that could alter the mtRFP localization, resulting in incorrect mitochondrial mass evaluation. For this reason, before proceeding to the flow cytometry readout, a rapid check of mtRFP intracellular localization by fluorescence microscopy is strongly recommended. Indeed, the use of cells stably-expressing mtRFP could also be useful to detect mitochondrial import defects. Conversely, most of the methods used to evaluate the mitochondrial mass (i.e., citrate synthase activity assay and mtDNA quantitation) could be affected by mitochondrial protein import defects, without any possibility to become aware of it. Moreover, another shortcoming of this method could be related to the clonal peculiarities of single clones. Clonal variability is a general concern of any study based on either modulation of protein expression or gene editing. However, selection of few clones (two or three) with similar phenotype, as we here reported choosing clones with similar mtRFP fluorescence intensity, is usually considered to be an effective approach to validate the data. The quantitative method we have developed would be easily adapted to high-throughput screening of novel drugs for enhancing or inhibiting autophagy/mitophagy and mitochondrial biogenesis, as for genes that regulate these processes. Future application of this analytical method will greatly benefit the understanding of autophagy/mitophagy and development of new drugs to treat autophagy/mitophagy-related disorders, including ageing [43] and cancer [44,45]. The method here presented is characterized by both high reliability and sensitivity to mitochondrial mass changes. However, its application is restricted to analysis of the mitochondrial mass changes within a selected cell line by using few clones exposed to different physico-chemical microenvironment conditions (drugs, oxygen tension, etc.). The main reason for this restriction is the variability among cell types in terms of transfection efficiency that in turn leads to a different plasmid copy number integrated in the cellular genome.

In conclusion, the use of cellular clones stably expressing mtRFP to evaluate the mitochondrial mass changes that occur in response to different experimental conditions provides the advantage of evaluating this parameter in intact cells and avoids artefacts due to biochemical and mechanical manipulation of cell samples.

## Abbreviations

BNIP3: BCL2/adenovirus E1B 19 kDa protein-interacting protein 3; BNIP3L: BCL2/adenovirus E1B 19 kDa protein-interacting protein 3-like; CS: citrate synthase; DMEM: Dulbecco’s Modified Eagle Medium; ΔΨ_m_: mitochondrial membrane potential; FUNDC1: FUN14 domain-containing protein 1; HBSS: Hank’s balanced salt solution; HIF-1α: hypoxia-inducible factor 1-α; mtDNA: mitochondrial DNA; mtFPs: mitochondrially targeted fluorescent proteins; mtRFP: mitochondrially targeted red fluorescent protein; MTS: mitochondrial targeting sequence; NAO: nonylacridine orange; OMM: outer mitochondrial membrane; OXPHOS: mitochondrial oxidative phosphorylation; PEI: polyethylenimine; PGC-1α or β: peroxisome proliferator-activated receptor gamma coactivator 1α or 1β; PINK1: PTEN induced protein kinase 1; RCR: respiratory control ratio; ROS: reactive oxygen species; RSV: resveratrol; TMRM: tetramethylrhodamine methyl ester; TOM: translocase of the outer membrane.
